# Disentangling relationships between Alzheimer's disease plasma biomarkers and established biomarkers in patients of tertiary memory clinics

**DOI:** 10.1016/j.ebiom.2024.105504

**Published:** 2024-12-18

**Authors:** Marina Bluma, Konstantinos Chiotis, Marco Bucci, Irina Savitcheva, Anna Matton, Miia Kivipelto, Andreas Jeromin, Giovanni De Santis, Guglielmo Di Molfetta, Nicholas J. Ashton, Kaj Blennow, Henrik Zetterberg, Agneta Nordberg

**Affiliations:** a Center of Alzheimer Research, Division of Clinical Geriatrics, Department of Neurobiology, Karolinska Institutet, Care Sciences and Society, Stockholm, Sweden; b Department of Neurology, Karolinska University Hospital, Stockholm, Sweden; c Karolinska University Hospital, Theme Inflammation and Aging, Stockholm, Sweden; d Turku PET Centre, Turku University Hospital, Turku, Finland; e Karolinska University Hospital, Medical Radiation Physics and Nuclear Medicine, Stockholm, Sweden; f Center of Alzheimer Research, Division of Neurogeriatrics, Department of Neurobiology, Karolinska Institutet, Care Sciences and Society, Stockholm, Sweden; g ALZPath, Inc, Carlsbad, CA, USA; h Department of Psychiatry and Neurochemistry, University of Gothenburg, Molndal, Sweden; i King's College London, Institute of Psychiatry, Psychology and Neuroscience Maurice Wohl Institute Clinical Neuroscience Institute London, UK; j NIHR Biomedical Research Centre for Mental Health and Biomedical Research Unit for Dementia at South London and Maudsley NHS Foundation London, UK; k Centre for Age-Related Medicine, Stavanger University Hospital, Stavanger, Norway; l Clinical Neurochemistry Laboratory, Sahlgrenska University Hospital, Mölndal, Sweden; m Department of Neurodegenerative Disease, UCL Institute of Neurology, Queen Square, London, UK; n UK Dementia Research Institute at UCL, London, UK; o Hong Kong Center for Neurodegenerative Diseases, Clear Water Bay, Hong Kong, China; p Wisconsin Alzheimer's Disease Research Center, University of Wisconsin School of Medicine and Public Health, University of Wisconsin–Madison, Madison, WI, USA

**Keywords:** Alzheimer's disease, Plasma biomarkers, Amyloid, Hyperphosphorylated tau proteins, pTau217

## Abstract

**Background:**

Several plasma biomarkers for Alzheimer's disease (AD) have demonstrated diagnostic and analytical robustness. Yet, contradictory results have been obtained regarding their association with standard diagnostic markers of AD. This study aims to investigate the specific relationship between the AD biomarkers currently used in clinical practice and the plasma biomarkers.

**Methods:**

In a memory clinic cohort, we analysed plasma pTau181, pTau217, pTau231, respectively, GFAP, NfL, CSF pTau181, Aβ-PET scans, and MRI/CT visual read of atrophy. We utilized methods based on multiple linear regression to evaluate the specific associations between clinically used and recently developed plasma biomarkers, while also considering demographic variables such as age and sex.

**Findings:**

Although plasma pTau181, pTau217, pTau231, and GFAP were significantly associated with both Aβ-PET and CSF pTau181, Aβ-PET explained more variance in the levels of these biomarkers. The effect of CSF pTau181 on plasma GFAP and pTau181 was completely attenuated by Aβ-PET, whereas pTau231 and pTau217 were affected by both Aβ-PET and CSF pTau181 levels. Unlike these biomarkers, increased NfL was rather indicative of brain atrophy and older age. Based on the effect sizes, plasma pTau217 emerged as highly effective in distinguishing between A+ and A−, and T+ and T− individuals, with 60% of variance in plasma pTau217 explained by clinical AD biomarkers.

**Interpretation:**

Amyloid burden primarily drives the changes in plasma pTau181, pTau217, pTau231, and GFAP. In contrast to plasma pTau217, a significant portion of variance in plasma pTau181, pTau231, GFAP, NfL remains unexplained by clinical AD biomarkers.

**Funding:**

This research is supported by the 10.13039/501100004359Swedish Research Council VR: 2017-06086, 2020-4-3018, 2024-2027; Swedish 10.13039/501100003792Brain Foundation, Swedish 10.13039/501100008599Alzhzeimer Foundation, CIMED Region Stockholm/10.13039/501100004047Karolinska Institutet; the Region Stockholm - 10.13039/501100004047Karolinska Institutet regional agreement on medical training and clinical research (ALF), Fondation Recherche sur Alzheimer (France).

**Research in context**

**Evidence before this study**

In research cohorts, plasma biomarkers of Alzheimer's disease (AD), including phosphorylated tau isoforms (pTau181, pTau231, pTau217) and Glial Fibrillary Acidic Protein (GFAP), have shown associations with amyloid and tau pathologies; whereas Neurofilament Light Chain (NfL) has been linked with neurodegeneration. It is important to understand how these findings translate into clinic settings and how changes in levels of these biomarkers are related to the results of current clinical tests.

**Added value of this study**

In a heterogeneous real-world clinical sample, our study demonstrated that various plasma biomarkers map differentially onto the AT(N) framework. Brain amyloid load, quantified with positron emission tomography (PET), emerged as a significant driver of changes in plasma biomarker levels. Additionally, we showed that the levels of unexplained variance in these plasma biomarkers significantly vary.

**Implications of all the available evidence**

Changes in plasma pTau181, pTau231, pTau217, and GFAP are associated with pathological changes typical of AD, yet they exhibit varying responsiveness to changes observed in amyloid PET or CSF pTau181. Among these biomarkers, plasma pTau217 outperforms the others. Plasma Neurofilament Light Chain (NfL), in contrast, has a distinct profile, providing information about the rate of brain atrophy rather than the core pathological processes in AD.

## Introduction

The abnormal accumulation of amyloid beta (Aβ), hyperphosphorylated tau proteins, and subsequent neurodegeneration are the core Alzheimer's disease (AD) pathologies.^1^^,^^2^ Presently, cerebrospinal fluid (CSF) measures of Aβ and tau levels, along with positron emission tomography (PET) imaging with Aβ and tau ligands, and MRI assessment of brain atrophy, are the established tools to support *in vivo* diagnosis of AD.^3^ While these markers are specific and clinically validated, they have significant limitations due to their high costs and invasiveness. Unrestricted by these constraints, recently developed plasma biomarkers have shown promise in identifying pathological features of AD.^4^

In research cohorts, plasma biomarkers have demonstrated high diagnostic sensitivity and specificity in detecting AD-type pathology.^5^, ^6^, ^7^, ^8^, ^9^, ^10^, ^11^ Among the emerging plasma biomarkers, glial fibrillary acidic protein (GFAP), a general marker of astroglial activation in response to neuronal damage occurring in neurodegenerative diseases,^12^ traumatic brain injury,^13^, ^14^, ^15^ acute stroke,^16^, ^17^, ^18^ or other CNS diseases,^19^ and tau phosphorylated at threonine 181 (pTau181), 217 (pTau217), and 231 (pTau231), have shown accurate and robust diagnostic performance discriminating between AD and non-AD pathology.^6^^,^^8^^,^^20^, ^21^, ^22^, ^23^ Despite the good accuracy in identifying individuals on the AD pathway, there is variability in findings regarding the specific neuropathological changes these biomarkers reflect.^24^, ^25^, ^26^ In the context of neurodegenerative diseases, blood GFAP levels has been reported to rise in response to both amyloid^20^ or tau pathology.^26^^,^^27^ In turn, one might intuitively associate plasma pTau with neurofibrillary tangles in the brain,^28^ although it has been shown that tau phosphorylated at specific sites might be rather reflective of amyloid pathology.^29^ Additionally, neurofilament light (NfL), a biomarker of neuroaxonal damage, has exhibited potential for the detection and monitoring of neuronal injury in multiple diseases.^30^, ^31^, ^32^ Notably, NfL was found to rise years before symptoms onset in autosomal dominant AD mutation carriers.^33^

The main aim of this study is to examine the specific relationships between plasma pTau181, pTau217, pTau231, GFAP, and NfL in comparison to biomarkers used in clinical practice for detecting AD-related pathologies (i.e., amyloid PET, CSF pTau181, and atrophy on MRI) in a cohort of patients of tertiary memory clinics. Additionally, we seek to quantify and compare the extent to which these biomarkers primarily reflect amyloid, tau, or both pathologies, after adjusting for the effects of demographic variables.

## Methods

### Participants

In this study, 138 participants from a tertiary memory clinical cohort were included retrospectively. All participants had been referred to the Clinic for Cognitive Disorders at Karolinska University Hospital Huddinge in Stockholm, Sweden, due to cognitive complaints by their primary care physicians (GPs) and, in hospital specialists or tertiary memory clinics for a second opinion. For this study we selected participants who had undergone an extensive clinical assessment at Karolinska Hospital between 2014 and 2019. This assessment included amyloid PET, CSF sampling, and blood draws. It is important to note that according to this protocol, only patients who underwent lumbar puncture also had their blood drawn. This protocol naturally limited our sample size, as it was contingent on the subset of patients who completed all aspects of this comprehensive diagnostic procedure. The sample covered a wide spectrum of conditions associated with cognitive or memory complaints, ranging from mild cognitive impairment (MCI) (both amyloid-negative (MCI Aβ-) and amyloid-positive (MCI Aβ+)), Alzheimer's disease (AD), non-AD dementia, and a small subset of individuals with subjective memory complaints (SMC). Information on patients' sex was obtained from their medical records, which are typically based on self-reported data.

### Diagnostic assessment

All participants underwent a comprehensive examination that included medical history, physical, neurological, psychiatric assessments, neuropsychological testing, CT or MRI, CSF biomarker analysis, and amyloid PET scan with either [^18^F]Flutemetamol or [^11^C]Pittsburgh compound-B (PiB). In patients of memory clinics, amyloid PET has demonstrated higher diagnostic accuracy compared to CSF Aβ42.^34^^,^^35^ The final diagnoses were reached through a consensus among a multidisciplinary dementia expert team, including specialists in cognitive disorders, clinical neuropsychologists, and specialist nurses.

The main diagnostic categories included MCI^36^; dementia due to Alzheimer's disease (ADD)^1^; and a non-AD dementia group with diagnoses of dementia of unclear aetiology^37^; Lewy body dementia,^38^ frontotemporal dementia^39^; vascular dementia including subcortical type^40^; primary age-related tauopathy^41^^,^^42^; and alcohol-related dementia.^43^

Amyloid PET images were visually assessed as positive or negative, and this assessment, combined with the available clinical information from the examination, led to the biomarker-based diagnoses: MCI Aβ– (n = 31), MCI Aβ+ (n = 29), ADD (n = 52), non-AD (n = 22), or SMC (n = 4). Of note, for the four patients in the SMC group, a diagnosis of a neurodegenerative disorder was ruled out, and no objective evidence of cognitive impairment were detected in this extensive clinical assessment.

### Plasma collection and analysis of plasma biomarkers

Blood was drawn under fasting conditions between 7 and 11 AM into sodium-heparin tubes (Vacutainer®, BD Diagnostics, catalog number 369623) and then centrifuged (1500×*g* (3000 rpm), +4 °C) for 10 min. Of note, the use of EDTA tubes for blood collection is more thoroughly validated in AD blood biomarker research. A study by Ashton et al.^44^ demonstrated that while various blood additives yield different absolute values, the results are highly correlated across different tube types. Therefore, as this study does not rely on specific cutoffs (either in general or derived from EDTA plasma), the pre-analytical factors should not affect the conclusions of the study. Following centrifugation, the samples were aliquoted into polypropylene tubes and stored at −80 °C within 30–60 min of collection. Pseudoanonymised samples were sent to the University of Gothenburg by temperature-regulated dry ice transport. Sample analyses were conducted in a blinded manner with respect to the diagnosis. Levels of plasma GFAP and NfL were quantified using a multiplexed single molecule array (SIMOA, N4PE from Quanterix). Plasma pTau181 and pTau231 were assessed using in-house developed SIMOA assays described in^5^ and,^45^ respectively. Plasma pTau217 were analysed using ALZpath Simoa assay (ALZpath INC).^23^

### PET imaging

[^18^F]Flutemetamol PET scans were acquired using either a Biograph mCT PET/CT scanner (Siemens/CTI) or GE Discovery scanner (General Electrics) at the Department of Medical Radiation Physics and Nuclear Medicine Imaging, Karolinska University Hospital, Huddinge, Sweden, whereas PiB scans were acquired at Uppsala PET centre on ECAT EXACT HR1 scanner (Siemens/CTI). PET scans were visually assessed as positive or negative by an experienced nuclear medicine physician (I.S.). Next, amyloid PET scans were pre-processed with the robust PET-only pipeline (rPOP)^46^ for PET-only datasets in MATLAB (MathWorks, v.R2022_a) and SPM 12, and Centiloids were calculated using an in-house Centiloid calibration pipeline, based on the methods described in^47^ and.^48^

### CSF collection and analysis

Samples of CSF were collected via standard lumbar puncture under non-fasting conditions. Sample analyses were performed at the Clinical Neurochemistry Laboratory, Sahlgrenska University Hospital, Mölndal, Sweden, where levels of pTau were determined using commercially-available sandwich ELISAs (Innogenetics). The CSF pTau181 levels were assessed against established cut-offs provided by the local laboratory that analysed the samples (≥80 pg/ml).

### MRI/CT

MRI and, in some cases, CT scans were visually evaluated using two rating scales: medial temporal atrophy^49^ and global cortical atrophy.^50^ Individuals were categorized as normal or abnormal on these scales using age-adjusted cut-offs.^51^^,^^52^ Those deemed abnormal on both scales were classified as N+ for analyses requiring dichotomous classification. When dichotomization was unnecessary, to account for the heterogeneity in atrophy patterns, the atrophy on MRI was classified as follows:1 (or N−, normal on both scales), 2 (or N+, abnormal on one scale), and 3 (or N++, abnormal on both scales). Two individuals lacked MRI or CT data and thus could not be classified according to the ATN system, resulting in 136 participants included in the biomarker comparisons among the ATN groups.

### AT(N) classification

Participants were classified according to the AT(N) system, which assesses each individual for the presence of amyloid deposition (A), tau aggregation (T), and neurodegeneration (N) based on amyloid-PET, CSF pTau181, and MRI/CT imaging, respectively.^1^ Our approach to classifying N through MRI/CT imaging deviates from the standard application of the NIA-AA research criteria and may have been stricter.

### Statistics

Statistical analysis was carried out in R version 4.3.2 (https://www.r-project.org), with data visualizations created using ggplot2 (v3.3.5).

The number of patients per group and their basic demographic and clinical characteristics can be found in Table 1. Baseline differences between the AT(N) groups were tested using Fisher's exact test for categorical variables, with the exception of clinical diagnosis and APOE genotype, for which the G-test was applied (‘DescTools’,v0.99.54). Differences in continuous variables were evaluated using the Kruskal–Wallis test (‘stats’, v4.3.4).

**Table 1:** Demographic characteristics and biomarker status across the ATN groups.

Characteristic	A−T−N−, N = 29^a^	A−T−N+, N = 27^a^	A+T−N−, N = 37^a^	A+T−N+, N = 12^a^	A+T+N−, N = 24^a^	A+T+N+, N = 7^a^	p-value^b^
Clinical diagnosis							<0.0001 (1)
MCI Aβ-	17/29 (59%)	14/27 (52%)	0/37 (0%)	0/12 (0%)	0/24 (0%)	0/7 (0%)	
MCI Aβ+	0/29 (0%)	0/27 (0%)	15/37 (41%)	2/12 (17%)	10/24 (42%)	2/7 (29%)	
AD	0/29 (0%)	0/27 (0%)	22/37 (59%)	10/12 (83%)	13/24 (54%)	5/7 (71%)	
Non-AD	8/29 (28%)	13/27 (48%)	0/37 (0%)	0/12 (0%)	1/24 (4.2%)	0/7 (0%)	
SMC	4/29 (14%)	0/27 (0%)	0/37 (0%)	0/12 (0%)	0/24 (0%)	0/7 (0%)	
Age (years)	64 (61, 70)	67 (62, 76)	63 (56, 68)	69 (64, 72)	64 (59, 70)	67 (65, 72)	0.069 (2)
Sex							0.0017 (3)
F	18/29 (62%)	8/27 (30%)	21/37 (57%)	4/12 (33%)	20/24 (83%)	5/7 (71%)	
M	11/29 (38%)	19/27 (70%)	16/37 (43%)	8/12 (67%)	4/24 (17%)	2/7 (29%)	
Aβ PET (CL)	−4 (−8, 5)	−6 (−18, −1)	86 (76, 103)	82 (68, 102)	79 (58, 111)	90 (83, 100)	<0.0001 (2)
CSF Aβ42	696 (574, 904)	725 (603, 832)	457 (408, 609)	504 (411, 553)	590 (547, 694)	673 (654, 689)	<0.0001 (2)
CSF pTau181	35 (25, 47)	38 (29, 45)	60 (49, 69)	49 (44, 67)	91 (84, 100)	100 (87, 125)	<0.0001 (2)
MMSE	27 (24, 29)	25 (22, 28)	27 (24, 28)	27 (23, 28)	27 (26, 29)	25 (22, 26)	0.090 (2)
Missing	0	1	2	0	0	0	
Tracer							0.012 (3)
[^11^C]PiB	4/29 (14%)	0/27 (0%)	10/37 (27%)	0/12 (0%)	3/24 (13%)	2/7 (29%)	
[^18^F]Flutemetamol	25/29 (86%)	27/27 (100%)	27/37 (73%)	12/12 (100%)	21/24 (88%)	5/7 (71%)	
APOE genotype							0.002 (1)
	0/20 (0%)	0/20 (0%)	1/30 (3.3%)	0/8 (0%)	0/16 (0%)	0/7 (0%)	
E2/E3	0/20 (0%)	1/20 (5.0%)	0/30 (0%)	0/8 (0%)	0/16 (0%)	0/7 (0%)	
E2/E4	1/20 (5.0%)	1/20 (5.0%)	0/30 (0%)	0/8 (0%)	0/16 (0%)	0/7 (0%)	
E3/E3	12/20 (60%)	15/20 (75%)	5/30 (17%)	1/8 (13%)	4/16 (25%)	1/7 (14%)	
E3/E4	3/20 (15%)	2/20 (10%)	15/30 (50%)	6/8 (75%)	7/16 (44%)	3/7 (43%)	
E4/E4	4/20 (20%)	1/20 (5.0%)	9/30 (30%)	1/8 (13%)	5/16 (31%)	3/7 (43%)	
Missing	9	7	8	4	8	0	

a n/N (%); Median (IQR).

b (1) G-test; (2) Kruskal–Wallis rank sum test; (3) Fisher's exact test.

The fluid biomarkers were log transformed accordingly (CSF pTau181, plasma GFAP, pTau181, pTau217, NfL) or square root transformed (pTau231). All subsequent analyses were performed using the transformed values.

To compare the AT(N) groups, we calculated the means and 95% confidence intervals (CIs) for each group. Group comparisons were based on the overlap of these 95% CIs; non-overlapping intervals were interpreted as indicative of significant differences between the group means. This analysis was not adjusted for age and sex because the sample size in each ATN groups (8–10 patients) was limited. However, in preliminary analysis, we did check for the effect of age, sex, and disease stage severity on each biomarker using one-way ANOVAs (Table S2).

Correlations between the (transformed) plasma biomarker values were assessed with Pearson correlations on the transformed biomarker values (except for amyloid PET and CSF Aβ 42) and age. Ordinal variables (MMSE, APOE, atrophy rate)–with Spearman correlation coefﬁcient and point-biserial for sex. These analyses were adjusted for age and sex by calculating the partial correlation coefficients (‘ppcor’, v1.1). To calculate the confidence intervals for partial correlation coefficients, we used a custom function that first computed the partial correlation between two variables while controlling for others using the ppcor::pcor function. Subsequently, we fit linear models to obtain residuals and conducted a correlation test on these residuals using the cor.test function to derive the confidence intervals (Table S3).

We then computed unstandardized difference between the means in plasma biomarker levels in the dichotomized groups (i.e., A+ vs A−, T+ vs T−, N+ vs N−), with bootstrapped 95% CIs (N = 10,000). To compare variance in plasma biomarkers across AT(N) groups we calculated a measure of spread by dividing interquartile range by the median (similar to the coefficient of variation) while remaining resistant to outliers. Next, we performed dominance analysis to compare the relative importance of the predictors. For each predictor, we present the weighted average of its contribution to an overall model fit by averaging results across all models in which the predictor is included (general dominance statistics). Age and sex were included to delineate the specific contribution of each biomarker towards the modulation of plasma biomarker levels.

A mediation analysis was conducted to assess if the plasma biomarkers were independently associated with core AD biomarkers (amyloid PET and CSF pTau181), or if the variance explained by one biomarker, which accounts for a smaller proportion of the variance, is mediated by another variable that explains a larger portion of the variance. In the meditation analysis, two regression models were specified: the mediator model predicting the amyloid PET from the independent variable, CSF pTau181; and the outcome model predicting the outcome (plasma biomarker) from the mediator (amyloid PET), and the independent variable (CSF pTau181). All models have been controlled for the effect of age and sex. The assumptions underlying the linear regression models were assessed in two ways: by visually inspecting the Q–Q plots and using the gvlma function (‘gvlma’, v1.0.0.3), which tests for skewness, kurtosis, heteroscedasticity, and link function. Then the mediate (‘mediation’, v4.5.0) function was then applied to the fitted mediator and outcome models to estimate the natural indirect (or average causal mediation) effect (ACME), average natural direct effect (ADE), and the total effect. The significance of direct and indirect effects was assessed using bootstrapping procedures, where unstandardized indirect effects were computed for each of 1000 bootstrapped samples, and the 95% confidence interval was computed by determining the indirect effects at the 2.5th and 97.5th percentiles. The collinearity of the models has been tested with the variance inflation factor, with a threshold of 5 was applied to identify problematic levels of collinearity.^53^ The regression models have been also assessed to meet assumptions of linear regression (skewness, kurtosis, link function and heteroscedasticity) (‘gvlma’, v1.0.0.3). The residuals of the models were inspected visually for homogeneity of variance and distribution using qq-plots. No model assumptions appeared violated.

### Ethics

Participants’ consent was obtained according to the Declaration of Helsinki. The Regional Human Ethics Committee of Stockholm, Sweden, and the Isotope Committee of Karolinska University Hospital Huddinge approved this study (Dnr 2009/816-41/4; Dnr 2011/1987-31/4; Dnr 2014-269-31; Dnr 2016/120-32, Dnr 2023-05898-02). All patients provided their written informed consent.

### Role of funders

The funding sources were not involved in the design and conduction of the study; collection, management, analysis, and interpretation of the data; preparation, review, or approval of the manuscript; or the decision to submit the manuscript for publication.

## Results

### Demographics

A total of 138 participants were included in this study. Their basic demographic characteristics are presented in Table 1. In brief, the average age was 65.2 years old, with a female-to-male ratio of 77–61. APOE genotyping was available in a subset of individuals (N = 102), of them 58.8% carriers of the APOE ε4 allele.

Classification according to the AT(N) scheme resulted in the presence of six ATN groups in our sample: A−T−N− (N = 29), A−T−N+ (N = 27), A+T−N− (N = 37), A+T+N− (N = 24), A+T−N+ (N = 12), and A+T+N+ (N = 7). The AT(N) groups were statistically different in terms of sex (p = 0.002, Fisher's exact test), with significantly higher presence of females in the A+T+N− group and more males in the A−T−N+ and A+T−N+ groups. The AT(N) groups also exhibited statistical differences in terms of the APOE ε4 genotype, with more ε4 non-carriers observed in A−T−N+ group and a higher count of ε4 carriers in all amyloid positive AT(N) groups. No significant differences were observed in terms of age or MMSE score. Please see Table 1 for details.

### Comparison of AT(N) groups

**Fig. 1:**
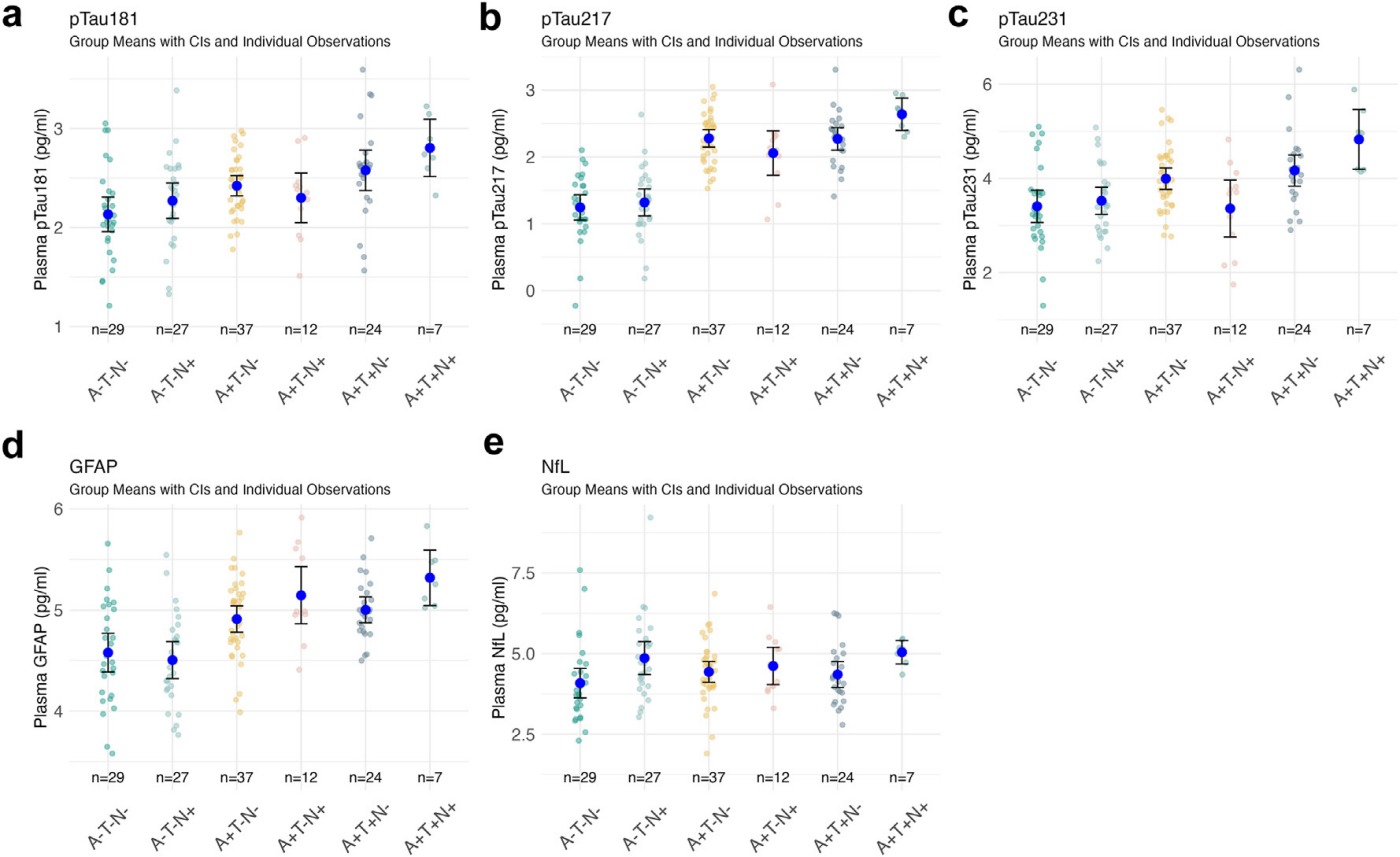
The levels of plasma biomarkers by the AT(N) group among n = 136 patients. Scatter plot showing mean differences with 95% confidence intervals CIs (error bars) across the AT(N) groups: (a). Levels of plasma pTau181 were higher in A+ patietns although there was overlap between the 95% CIs, (b). Plasma pTau217 showed clear differences between the means of all A+ groups and all A− groups with not overplapping 95% CIs, (c). Level of plasma pTau231 was higher in A+ groups with the highest concentrations observed in the A+T+N± groups, (d). Plasma GFAP levels increased progressively with higher pathology load, and (e). With plasma NfL did not vary significantly across the AT(N) groups, although compared to A−T−N−, levels of plasma NfL were higher in A−T−N+ and A+T+N+ groups. Not adjusted for age and sex.

Differences in means were observed across the AT(N) groups for all plasma biomarkers, with the largest difference observed for plasma ptau217 and the smallest—for plasma NfL. Specifically, plasma pTau181 levels were higher in amyloid positive groups compared to amyloid negative groups, although the 95% CIs overlapped (Fig. 1a). In contrast, plasma pTau217 showed clear difference between the means and the 95% CIs of the amyloid negative vs amyloid positive AT(N) groups, displaying the narrowest 95% CIs observed (Fig. 1b). Plasma pTau231 demonstrated differences between the means of most of the amyloid negative vs amyloid positive groups. Additionally, notably higher levels of plasma pTau231 were observed in the A+T+N+ and A+T+N− groups compared to the A+T−N+ group (Fig. 1c). The group means in plasma NfL levels differed between groups with and without neurodegeneration (N− and N+) but no AD-related pathology (A−T−), although in general changes in plasma NfL appeared less pronounced and characterised by overlapping 95% CIs (Fig. 1e). The levels of plasma GFAP seems to increase stepwise in amyloid positive individuals with the pathology exacerbation (Fig. 1d).

As evident from the scatterplot and the bar plot of the coefficient of variation (Figure S2), among the plasma biomarkers in the AT(N) groups, GFAP values displayed the widest range of variation. This wide spread of the plasma GFAP values, among amyloid negative individuals, did not allow a distinct visual separation between the amyloid-negative and amyloid-positive AT(N) groups, which was observed with plasma pTau217 (Fig. 1b, d).

### Plasma biomarkers in relation to other clinical and demographic variables

**Fig. 2:**
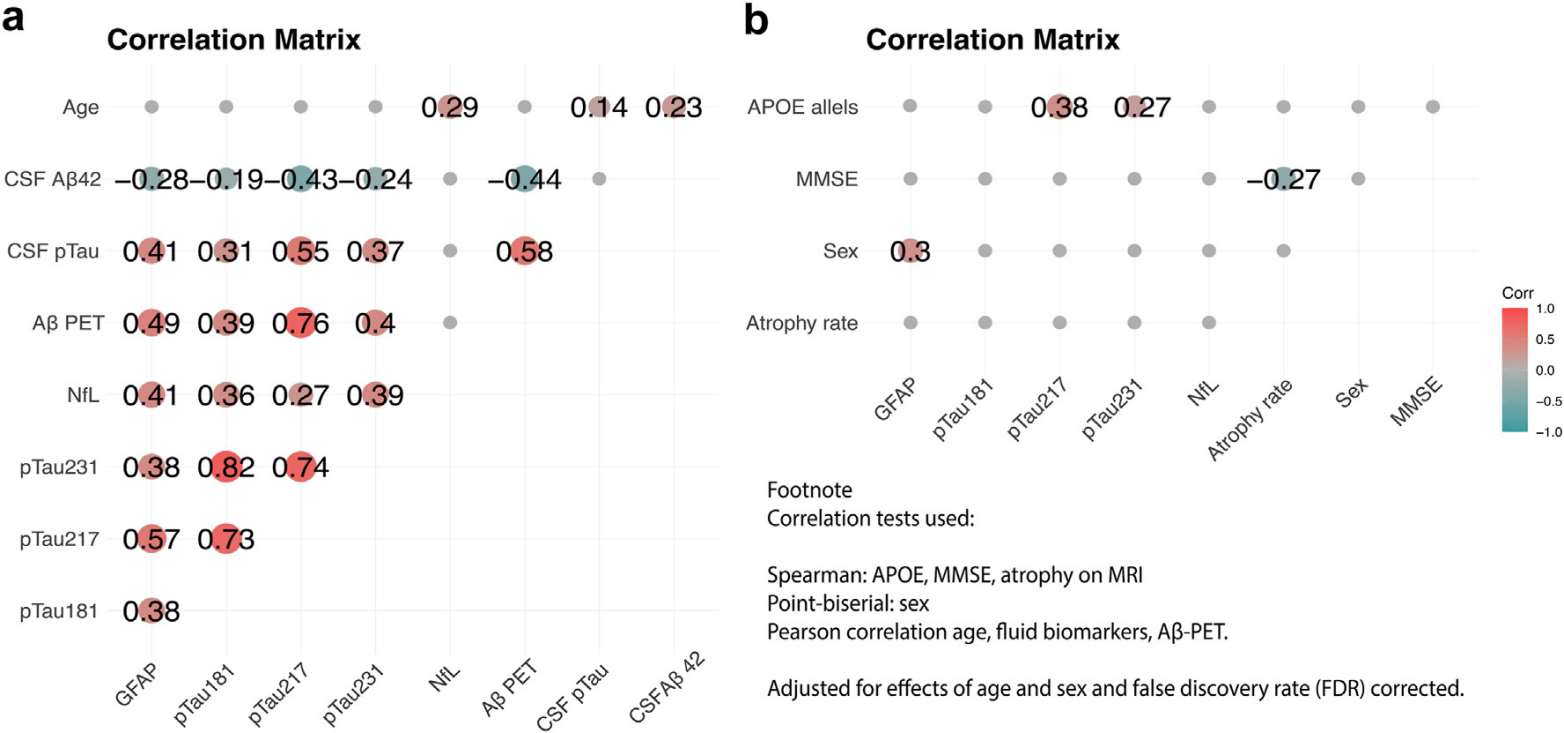
Relationship between plasma biomarkers and clinical and demographic variables among n = 138 patients: positive correlations (r/ρ > 0) are shown in red, while negative correlations (r/ρ < 0) are shown in blue. (a). Correlation matrix showing the Pearson's *r* coefﬁcients for the associations between the continuous variables considered. Due to the non-normal distribution, variables were transformed. (b). The correlation matrix showing the Spearman's *rho* coefﬁcients for the associations between the ordinal variables considered. Results were adjusted for the effect of age and sex, and FDR corrected.

A correlation matrix was generated to visualize the correlation coefficients between the plasma biomarkers, the clinical AD biomarkers (CSF Aβ42, CSF pTau181, MMSE, Atrophy on MRI, Aβ-PET (CL)) (Fig. 2a and b), and the demographic variables/AD risk factors (age, sex, number of APOE-ε4 alleles) (Fig. 2b). The analysis revealed that among all associations, the strongest positive correlation with the narrowest confidence interval was observed between plasma pTau217 and Aβ-PET (*r* = 0.76, 95% CI: 0.7, 0.8), followed by plasma GFAP and Aβ-PET (*r* = 0.49, 95% CI: 0.4, 0.58). Moderate positive correlations were also observed for plasma pTau181 and pTau231 with Aβ-PET, with coefficients of *r* = 0.39 (95% CI: 0.29, 0.49), *r* = 0.40 (95% CI: 0.29, 0.49), respectively. Similarly, moderate positive correlations were found between CSF pTau181 and plasma biomarkers, with a strongest correlation again for pTau217 (*r* = 0.55, 95% CI: 0.47, 0.63), followed by GFAP (*r* = 0.41, 95% CI: 0.3, 0.5), pTau231 (*r* = 0.37, 95% CI: 0.26, 0.47), and pTau181 (*r* = 0.31, 95% CI: 0.2, 0.41). Moderate negative correlations were observed between CSF Aβ-42 and the plasma biomarkers. Notably, the highest negative correlation was recorded for pTau217 (*r* = −0.43, 95% CI: −0.52, −0.32), followed by pTau231 (r = −0.24, 95% CI: −0.35, −0.13), GFAP (*r* = −0.28, 95% CI: −0.39, −0.17), and pTau181 (*r* = −0.19, 95% CI: −0.31, −0.8). Of note, plasma NfL did not correlate with the amyloid or CSF pTau181 biomarkers. It had a positively correlation with MRI/CT atrophy (*r* = 0.21, 95% CI: 0.06, 0.37), although no longer significant after adjustment for the effect of age (*r* = 0.14, 95% CI: −0.05, 0.29). Finally, all plasma pTau isoforms were highly intercorrelated (*r* ∈(0.73–0.82, 95% CI: 0.67–0.78, 0.78–0.85)). Additionally, Aβ-PET and CSF pTau181 were found to be intercorrelated, *r* = 0.58 (95% CI: 0.5, 0.66; Fig. 2a).

The negative association between the amyloid PET load (CL) and plasma pTau217 with atrophy on MRI may be explained by group imbalances: both the N− and N+ groups were notably enriched by amyloid-positive individuals (*χ*^*2*^ *test*, p = 0.009, Table S1). However, when examining the differences in atrophy rates, amyloid, and plasma pTau217 separately within amyloid-negative and -positive groups, this effect was not observed (Figure S1).

Among all plasma biomarkers, NfL exhibited the strongest association with age (ρ = 0.30). Female sex was positively associated with GFAP (ρ = 0.23), and APOE was correlated with plasma pTau217 and pTau231 (ρ = 0.41, ρ = 0.26), as shown in Fig. 2b.

### Unstandardized difference between the means

**Fig. 3:**
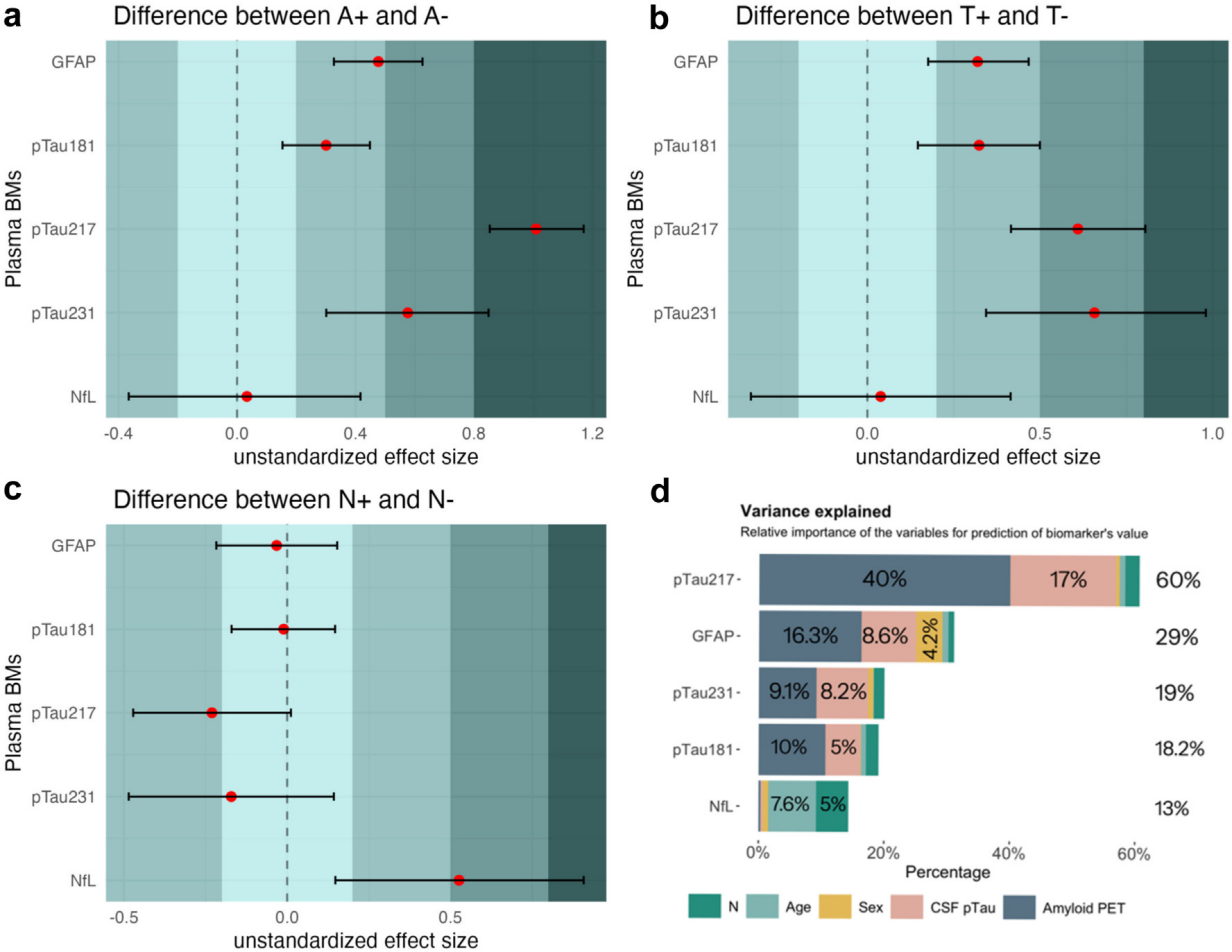
The forest plot illustrating unstandardized mean difference along with 95% bootstrapped confidence intervals (error bars) (a–c), estimated for the plasma biomarkers between A+/A−, T+/T−, and N+/N− groups in 138 individuals (136 in panel (c)); and the stacked bar plot displaying the results of dominance analysis. (a). Plasma GFAP and pTau217 had greater difference between the means and narrower 95% CI when differentiating between A+/A−; (b). Plasma pTau231 and pTau271 had greater mean difference when differentiating between T+/T−; (c). For the N+/N− group comparison, plasma NfL showed the largest, although modest, mean difference and a wide 95% CI. (d). Results of the dominance analysis in terms of variance explained. The names of the plasma biomarkers are displayed on the y-axis, and the corresponding percentage of variance explained illustrated in the coloured segments of the bars. Each colour fill represents a specific variable, as defined in the plot legend.

Next, we compared the unstandardized difference between the means of the plasma biomarkers to determine which ones exhibited a larger difference between groups with and without the presence of amyloid (A), tau (T) pathology, and neurodegeneration (N), in separate analyses for each of the AT(N) modalities. Our findings indicated that plasma pTau217 and GFAP demonstrated a larger difference between the means when comparing groups with amyloid (A+) and without amyloid (A−) (Fig. 3a). Plasma pTau231 and pTau217 showed a greater difference between the means of groups with tau pathology (T+) and without tau pathology (T−), as depicted in Fig. 3b, although the overlap in the 95% CI between plasma pTau181, 217, 231 and GFAP suggests a potential similarity in difference between the means. Lastly, plasma NfL exhibited the largest mean difference between the N+ and N− groups relative to other plasma biomarkers, as illustrated in Fig. 3c. However, its performance was moderate compared to the mean difference in pTau217 between individuals with amyloid pathology (A+) and those without (A−) and characterised by wider uncertainty in its measurement was observed.

### Relative contribution of established AD biomarkers and demographic variables in explaining the variance of plasma biomarkers

The dominance analysis was conducted to evaluate the specific contributions of amyloid PET and CSF pTau181 to each plasma biomarker level using standardized coefficients (SC). Amyloid PET exhibited the greatest relative importance in explaining the variance of plasma GFAP and pTau isoforms, ranging from 40% for pTau217 to 10% for pTau181, followed by CSF pTau181, ranging from 17% for pTau217 to 5% for pTau181 (Fig. 3d). Although, demographic variables such as age and sex explained up to 0.8% of the variance in pTau isoforms, sex accounted for 4.2% of the variance in plasma GFAP, while age contributed up to 7.6% to plasma NfL levels. Of note, this was the largest contributor that explained the variance in levels of plasma NfL, followed by atrophy on MRI/CT (denoted as N, up to 5%). Overall, the proportion of variance in the investigated plasma biomarkers explained by core AD biomarkers and demographic variables varied significantly, ranging from up to 60% for plasma pTau217 to only 13% for plasma NfL.

### Amyloid PET mediates the effect of CSF pTau181 on plasma biomarkers

**Figure 4:**
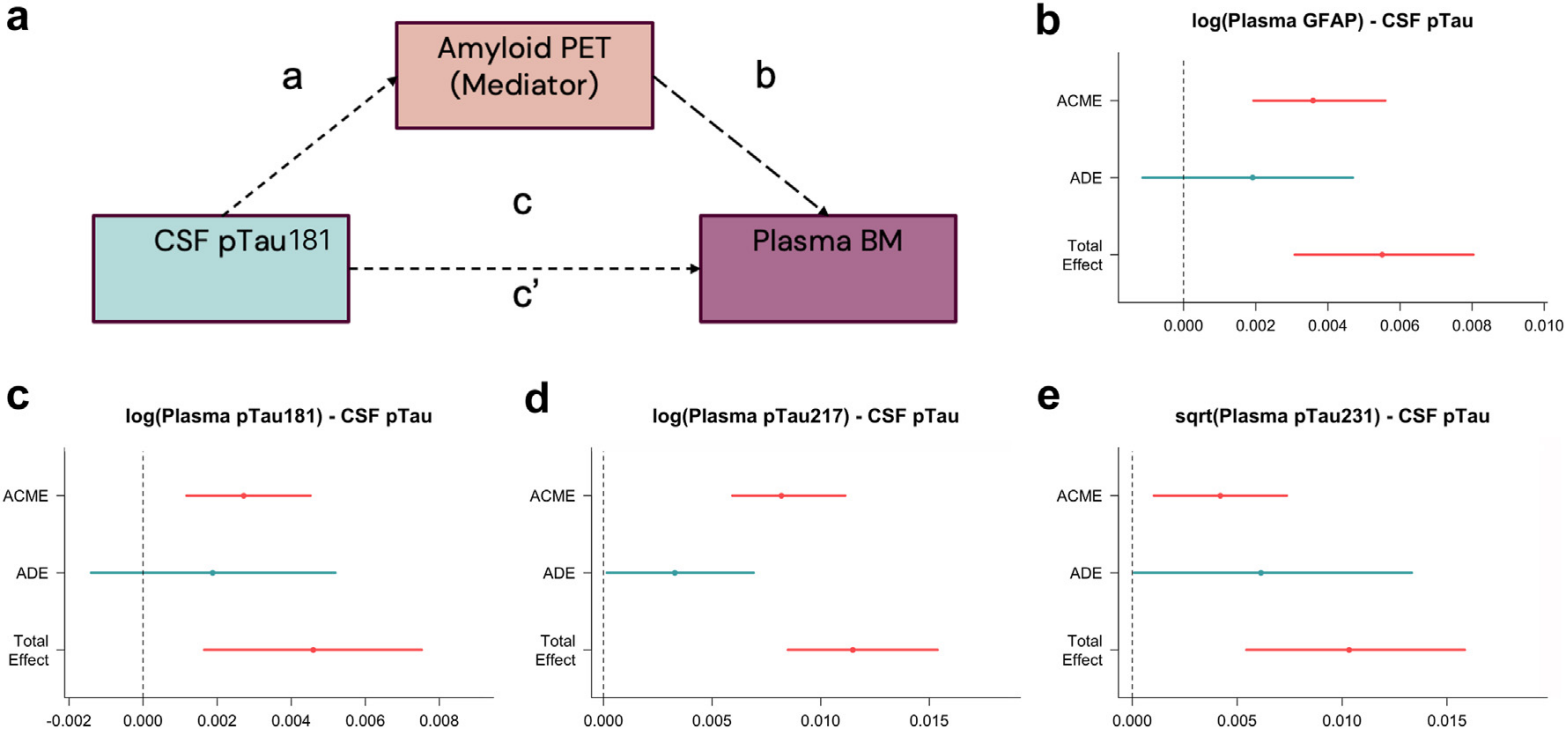
(a). The mediation path diagram illustrating the interactions between three biomarkers: Amyloid PET (Mediator), CSF pTau181, and plasma biomarkers as the outcome among n = 138 patients. ab (ACME) = Average Causal Mediation Effect of amyloid PET, c (ADE) = Average Direct Effect of CSF pTau181, c’ = Direct Effect after accounting for the indirect effects. b–e. Results of the mediation analysis. (b and c). The effect of CSF pTau181 on plasma GFAP and pTau181 was fully mediated via amyloid PET. (d and e). However, for plasma pTau231 and pTau217 values were only partial mediated via amyloid PET, with CSF pTau181 also showing a direct effect. The error bars show 95% confidence intervals (CI).

To investigate whether the established clinical biomarker (amyloid PET) might explain the largest portion of variance in plasma biomarkers but also mediate the effects of other correlated variables (CSF pTau181), we conducted a mediation analysis. Of note, assumption of the linear regression models on which the mediation analysis was based do not appear violated. The results revealed that for plasma GFAP and pTau181, the effect of CSF pTau181 was fully mediated by amyloid PET, with a average direct effect of CSF pTau181 that was insignificant (Fig. 4b and c). Conversely, the effects of CSF pTau181 on plasma pTau217 and pTau231 demonstrated both a direct and indirect path being significant (p < 0.0001, based on 1000 bootstrapped samples, with unstandardized indirect effects and 95% CI), suggesting that while part of the effect could be attributed to amyloid PET, CSF pTau181 also could exhibit some direct influence on the levels of these two plasma biomarkers (Fig. 4d and e).

## Discussion

The current study focuses on understanding the variability in levels of plasma pTau181, pTau217, pTau231, GFAP, and NfL in relation to the clinically used biomarkers of AD (i.e., amyloid-PET, CSF pTau181, and brain atrophy on MRI) in a cohort of patients of memory clinics. The novelty of this study stems from the observation that earlier studies on plasma biomarkers have mostly been done in well-controlled research-based settings and in populations with low heterogeneity and limited comorbidities.^54^ We observed that higher levels of pTau181, pTau217, and pTau231, and GFAP were associated with elevated levels of the core clinical pathophysiological AD biomarkers, especially with increased amyloid PET burden.

Interestingly, all measured plasma pTau isoforms correlated with amyloid PET, which highlights the role of amyloid in either tau hyperphosphorylation^55^^,^^56^ and/or in the subsequent release of tau residues into plasma and is in line with earlier suggestions that tau might become hyperphosphorylated in response to amyloid.^57^ Of note, the association between higher amyloid deposition and Blood–Brain-Barrier dysfunction in AD had been previously reported and may contribute to a mechanistic explanation for the amyloid associated release of pTau isoforms into the circulatory system (for a review see^58^).

The effect of CSF pTau181 on plasma GFAP and pTau181 was not significant after adjusting for amyloid PET. This finding aligns with a previous PET study of plasma GFAP, where after adjusting for amyloid, the association with tau PET was no longer significant.^59^ This, together with earlier studies,^20^^,^^24^^,^^60^ suggests that plasma GFAP could act as a biomarker for brain amyloid-β rather than tau pathology in AD. However, acknowledging the contradictory results from post-mortem^26^ and PET studies^61^ alongside the non-specific nature of GFAP,^62^^,^^63^ it is plausible that GFAP levels may reflect different pathologies depending on the stage of the disease: amyloid pathology at the early stages and tau pathology at the later stages.

In contrast to plasma pTau181, both plasma pTau217 and pTau231 showed a significant correlation with both CSF pTau181 and amyloid PET. In line with our observations, earlier studies have reported that plasma pTau217 correlates strongly with CSF pTau181 isoforms in head-to-head comparisons of plasma p-tau assays.^64^^,^^65^ Unlike plasma pTau217, the correlation between CSF and plasma pTau181 typically ranged from weak to moderate, as previously reported.^64^, ^65^, ^66^, ^67^ Corroborating these findings, our study supports the notion that plasma pTau217 exhibits a stronger association with post-translational modifications of pTau truncated at various threonine residues, suggesting that pTau217 is more effectively translated or detected in blood compared to other pTau isoforms. Taken together, our findings align with recent reports that pTau217 is associated with both Aβ and tau tangle aggregation in symptomatic individuals.^23^^,^^68^, ^69^, ^70^, ^71^

Our results suggest that pTau231 offers insights into the levels of CSF pTau181, the established clinical marker of tau pathology within the AT(N) biomarker framework to date. These findings are also consistent with *in vivo* studies using amyloid and tau PET imaging^24^^,^^70^^,^^71^ which demonstrate that both pTau231 and pTau217 levels correlate with the presence of tau neurofibrillary tangles in individuals with cognitive impairments. Additionally, these results also agree with post-mortem analyses demonstrating that elevated levels of plasma pTau231 are indicative of higher Braak stages.^24^ This is further supported by an *in vivo* PET study, showing that plasma pTau231 concentrations are different between stage 0 by PET-based Braak stage II.^72^ Moreover, plasma pTau231 performance has also been shown to be superior to pTau217 performance in discriminating between A+T+ and A−T− groups.^25^ Given, that pTau231 seems to show an earlier increase than pTau217 in samples enriched with cognitively unimpaired individuals, it does not, however, rise significantly with further exacerbation of amyloid pathology.^10^ The observation of an association between CSF pTau181 and pTau231 suggests that tau, rather than amyloid pathology, may contribute to the subsequent elevation of pTau231 levels.

The association of NfL with age and brain atrophy on MRI/CT, observed in our study, aligns with those documented in previous studies.^73^, ^74^, ^75^, ^76^, ^77^, ^78^ Of note, plasma NfL was the strongest predictor of conversion from MCI to AD dementia.^79^ This further underscores the potential of NfL to serve as a marker in the advanced stages of the disease, rather than as an early marker of core pathological changes in sporadic AD.

Notably, over 50% of the variance in plasma pTau181, pTau231, GFAP, and NFL remained unexplained by demographic and clinical variables. Previous research indicates that plasma AD biomarkers are influenced by various factors, including comorbidities (e.g., ischemic heart disease, cerebrovascular disease, chronic kidney disease, hyperlipidemia), lifestyle factors (smoking, alcohol consumption), body mass index (BMI), moderate-to-severe white matter hyperintensities, medication, and even personality traits.^80^, ^81^, ^82^, ^83^, ^84^, ^85^, ^86^, ^87^, ^88^ This underscores the importance of cautious interpretation of these biomarkers, especially when coming from a less well characterised cohorts of older age individuals, where comorbidities are highly prevalent.^89^

In conclusion, the findings from the current study, as well as those previously reported, demonstrated that plasma pTau217 closely correlates with both amyloid and tau pathology and is characterised by lower uncertainty in its measurement compared to other plasma biomarkers. Plasma pTau231 also correlates with both pathologies, albeit to a lesser extent, whereas pTau181 primarily serves as a marker for amyloid pathology. However, the precise mechanisms underlying these differential associations remain unclear and warrant further exploration. Specifically, it is unclear how contributions from a particular pTau epitope (T181, T217, T231), the biochemical properties of assays, and/or the accuracies of measurement platforms influence these relationships.^65^ Plasma GFAP also demonstrates a strong association with amyloid pathology at the early disease stage, suggesting that it is a marker associated with the neuroinflammatory response to initial amyloid accumulation. Overall, the four proteins measured in plasma appear to exhibit a similar biomarker profile, with plasma pTau217 most closely associated with the biomarkers of amyloid and tau pathology used in clinic, and standing out as the superior biomarker for AD. As expected, NfL appeared to have less predictive value in a cohort of patients enriched with AD; however, it seemed to provide some insights about brain atrophy.

### Limitations

A subset of patients included in the study were referred to Aβ PET investigations because their CSF biomarker profile did not provide confidence in diagnosing a patient as being either amyloid-negative or amyloid-positive. As a result, our sample may have been enriched with cases that are diagnostically more challenging. Another limitation of this study could be the use of CSF pTau181 as a marker of tau pathology, as some research suggests that tau-PET may be a more accurate marker of tau pathology. However, tau-PET is not currently included in clinical workups, and its availability in purely clinical dataset is limited. Also, we acknowledge that the small sample size of the present study may limit the generalizability of the findings. In particular, the small sample size for two of the resulting ATN profiles warrants cautious interpretation of the results. Future studies with larger sample sizes are needed to confirm these results and further our understanding of the difference between the plasma biomarkers across the ATN profiles. Another limitation is that we could not account for several important confounders known to influence the levels of plasma biomarkers, such as BMI, kidney function, medication, etc. High levels of unexplained variance attributable to uncontrolled factors highlight the need for future studies to include a comprehensive panel of potential confounders to improve the accuracy and reliability of biomarker measurements. Nonetheless, we have been able to investigate plasma biomarkers in a realistic uncontrolled clinical setting at a tertiary memory clinic, with a heterogeneous cohort of patients referred from different clinical practice. The patients were checked to ensure no major disturbances in renal function, but other common comorbidities in this clinical cohort, such as previous stroke, myocardial infarcts, cancer, and medications, could potentially influence the observations.

## Contributors

Study conceptualization and design, MBlu, KC, MBu, AN; Data curation, acquisition, and analysis, MBlu, MBu, IS, NJA, KC, AM, MK, AJ, GDS, GDM, KB, HZ, AN; Administrative, technical, or material support: NJA, AJ, HZ, AN. Writing—original draft, MBlu, AN; Writing—review & editing, MBlu, KC, MBu, AN, and all authors revised. MBlu, KC, MBu, and AN—had access to the data and verified it. All authors read and approved the final version of the manuscript.

## Data sharing statement

No data from this study has been deposited in external repositories or made publicly available, in order to maintain the privacy of research participants. However, anonymized data can be shared upon reasonable request by a qualified academic investigator, solely for the purpose of replicating the procedures and results presented in this article. Data sharing is subject to compliance with local legislation and approval by the local Ethical Review Board overseeing the cohort. A formal material/data transfer agreement must also be established before any data transfer occurs. Standard R libraries were used to analyse the data. Code can be shared upon request.

## Declaration of interests

Agneta Nordberg has received consulting fees from Lundbeck AB, Hoffman La Roche, AVVA Pharmaceuticals, has also given lectures in symposia organized by Hoffman La Roche; has participated in data safety monitoring or advisory board in Dementia Platform UK; has acted as a Deputy chairman in Wenner-Gren Foundation, Sweden.

Nicholas J. Ashton has served as consultant for Quanterix and has given lectures in symposia sponsored by Alamar Biosciences, Biogen, Eli-Lilly, and Quanterix; has also been granted a patent application (No.: PCT/US2024/037834 (WSGR Docket No. 58484-709.601 (‘Methods for Remote Blood Collection, Extraction and Analysis of Neuro Biomarkers’).

Henrik Zetterberg has served on scientific advisory boards and/or as a consultant for AbbVie, Acumen, Alector, Alzinova, ALZPath, Amylyx, Annexon, Apellis, Artery Therapeutics, AZTherapies, Cognito Therapeutics, CogRx, Denali, Eisai, Merry Life, Nervgen, Novo Nordisk, Optoceutics, Passage Bio, Pinteon Therapeutics, Prothena, Red Abbey Labs, reMYND, Roche, Samumed, Siemens Healthineers, Triplet Therapeutics, and Wave, has given lectures in symposia sponsored by Alzecure, Biogen, Cellectricon, Fujirebio, Lilly, Novo Nordisk, and Roche; is chair of the Alzheimer's Association Global Biomarker Standardization Consortium, and is a co-founder of Brain Biomarker Solutions in Gothenburg AB (BBS), which is a part of the GU Ventures Incubator Program.

Kaj Blennow has served on scientific advisory boards and/or as a consultant for AbbVie, AriBio, ALZpath, BioArctic, AC Immune, Biogen, Eisai, Lilly, Neurimmune, Ono Pharma, Prothena, Roche Diagnostics, Siemens Healthcare, has produced/participated in educational programs with Biogen, Eisai, Roche Diagnostics, has participated in advisory boards of Julius Clinical and Novartis, is a co-founder of Brain Biomarker Solutions in Gothenburg AB (BBS), which is a part of the GU Ventures Incubator Program.

Konstantinos Chiotis receives consultancy fees from LOGEX.

Miia Kivipelto has served as a member at scientific advisory boards and/or as a consultant of Combinostics, BioArctic, Eli Lilly, and Nestle; has given lectures in symposia sponsored by Elsai, Novo Nordisk, Nutricia, and Roche, has served in the board of Governors of the Alzheimer's Drug Discovery Foundation and as a member of World Dementia Council.

Andreas Jeromin has been an employee of ALZpath, Inc.
