# Supplementary material for: Disentangling relationships between Alzheimer's disease plasma biomarkers and established biomarkers in patients of tertiary memory clinics
Source: eBioMedicine. 2024 Dec 18;112:105504. doi: 10.1016/j.ebiom.2024.105504 (PMC11873569; doi:10.1016/j.ebiom.2024.105504)
Supplement: Supplementary Figures and Tables [file mmc1.docx]

**Supplement material**


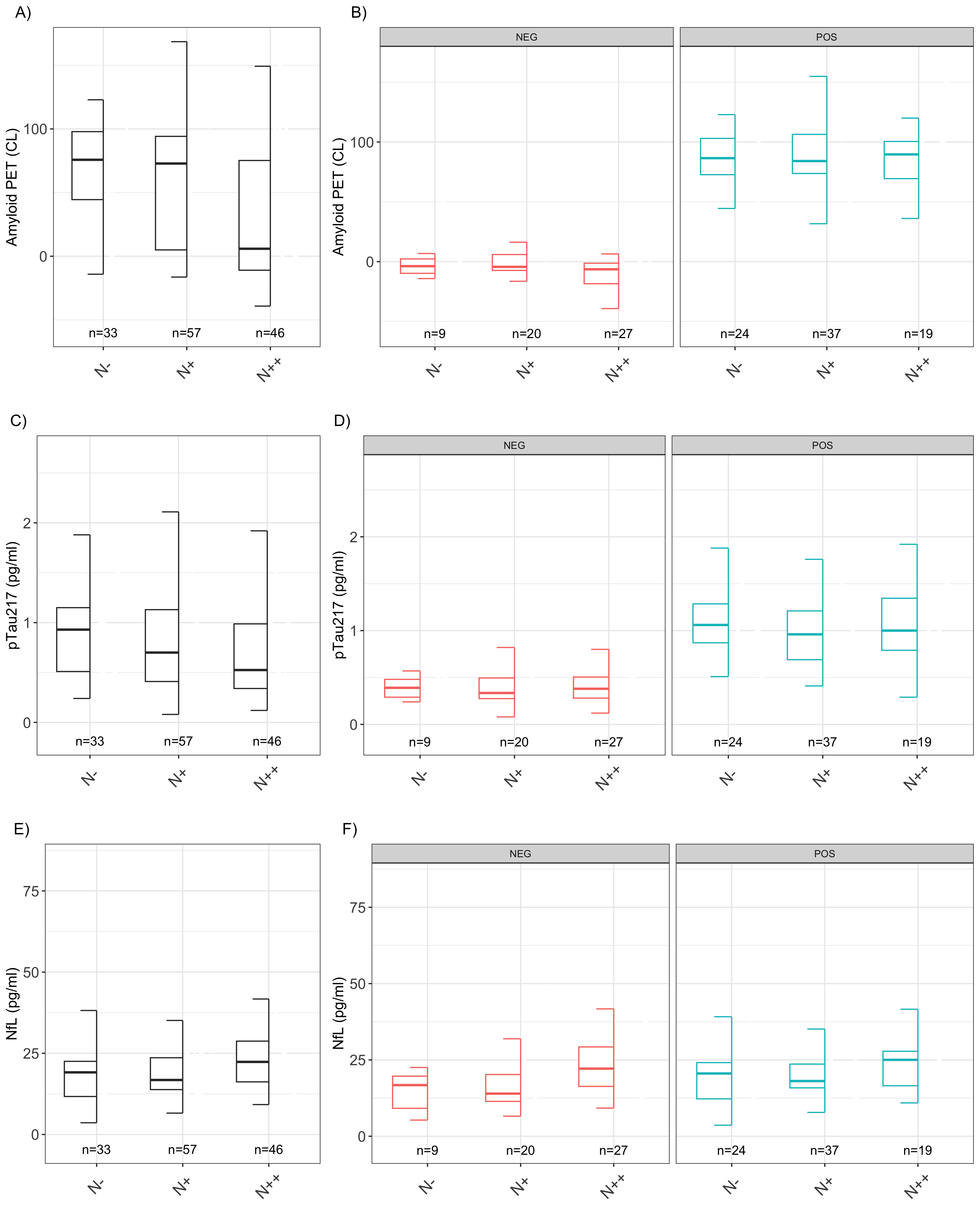


**Figure S1.** The association between neurodegeneration and amyloid PET (CL), plasma pTau217, and NfL in the whole sample, amyloid positive and negative individuals. **(A)** In the whole sample, patients with more advanced neurodegeneration (N++) had lower amyloid PET burden. However, when comparing amyloid negative and positive groups separately, the levels of amyloid load remained consistent across groups with different neurodegeneration statuses **(B)**. Note the imbalance in group sizes: N- group contained 24 amyloid positive compared to 9 amyloid negative individuals. **(C)** In the entire sample, patients with more advanced neurodegeneration tended to have lower levels of plasma pTau217. However, when examining amyloid positive and negative groups separately, the plasma pTau217 levels were on the same level across groups with different neurodegeneration statuses **(D)**. **(E)** In the entire sample, the subgroup of patients with more advanced neurodegeneration had higher plasma NfL. However, in separate amyloid positive and negative groups this effect was small **(F)**.

In the box, the top horizontal line represents Q1, the bold horizontal line inside the box indicates median, and the bottom of the box – Q3. The whiskers extending from the box indicate variability outside the Q1 and Q3, up to 1.5 times the interquartile range (IQR), respectively.

**Table S1.** Demographic and clinical characteristics of the N-, N+, and N++ groups. Both the N- and N+ groups were enriched by amyloid-positive individuals.

|  | N- (N=33) | N+ (N=57) | N++ (N=46) | Total (N=136) | *P*-value |
| --- | --- | --- | --- | --- | --- |
| **FLUTE_SUVR** | |  |  |  | 0.009 (1) |
| NEG | 9 (27.3%) | 20 (35.1%) | 27 (58.7%) | 56 (41.2%) |  |
| POS | 24 (72.7%) | 37 (64.9%) | 19 (41.3%) | 80 (58.8%) |  |
| **Age** |  |  |  |  | 0.007 (2) |
| Mean (SD) | 62.9 (5.4) | 64.6 (8.7) | 68.2 (8.5) | 65.4 (8.2) |  |
| Min - Max | 54.3 - 75.0 | 44.0 - 85.0 | 42.0 - 86.0 | 42.0 - 86.0 |  |
| **Sex** |  |  |  |  | 0.006 (1) |
| F | 21 (63.6%) | 38 (66.7%) | 17 (37.0%) | 76 (55.9%) |  |
| M | 12 (36.4%) | 19 (33.3%) | 29 (63.0%) | 60 (44.1%) |  |
| **MMSE** |  |  |  |  | 0.007 (2) |
| Mean (SD) | 27.1 (2.6) | 25.7 (3.6) | 24.7 (3.5) | 25.7 (3.5) |  |
| Min - Max | 20.0 - 30.0 | 16.0 - 30.0 | 17.0 - 30.0 | 16.0 - 30.0 |  |

*1. Pearson's Chi-squared test*

*2. Kruskal-Wallis rank sum test*

*
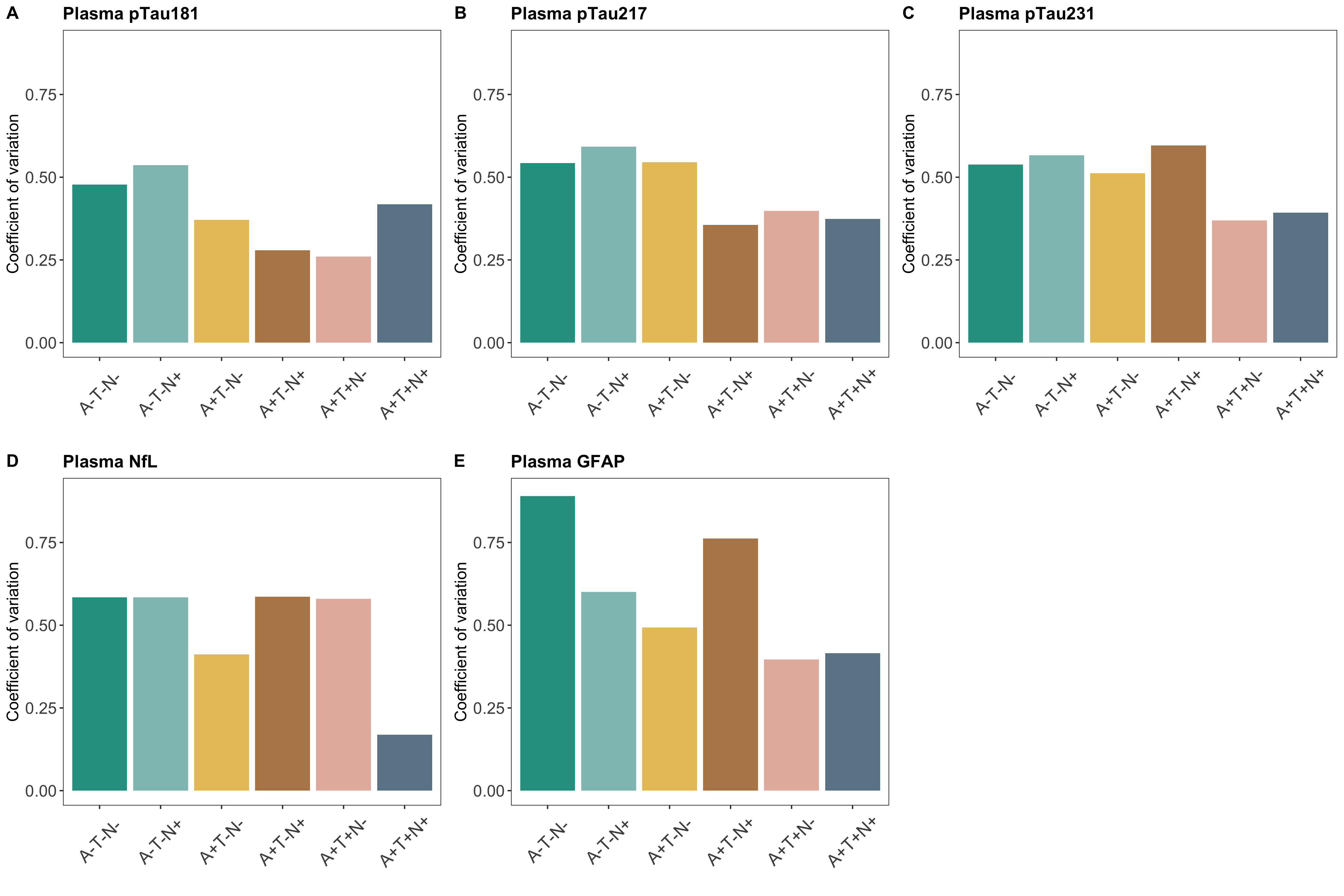
*

**Figure S2.** Coefficients of variation obtained by dividing interquartile ranges by the medians:

**(A)**. pTau181, **(B)**. pTau217, **(C)**. pTau217, **(D)**. NfL, and **(E)**. GFAP.

*
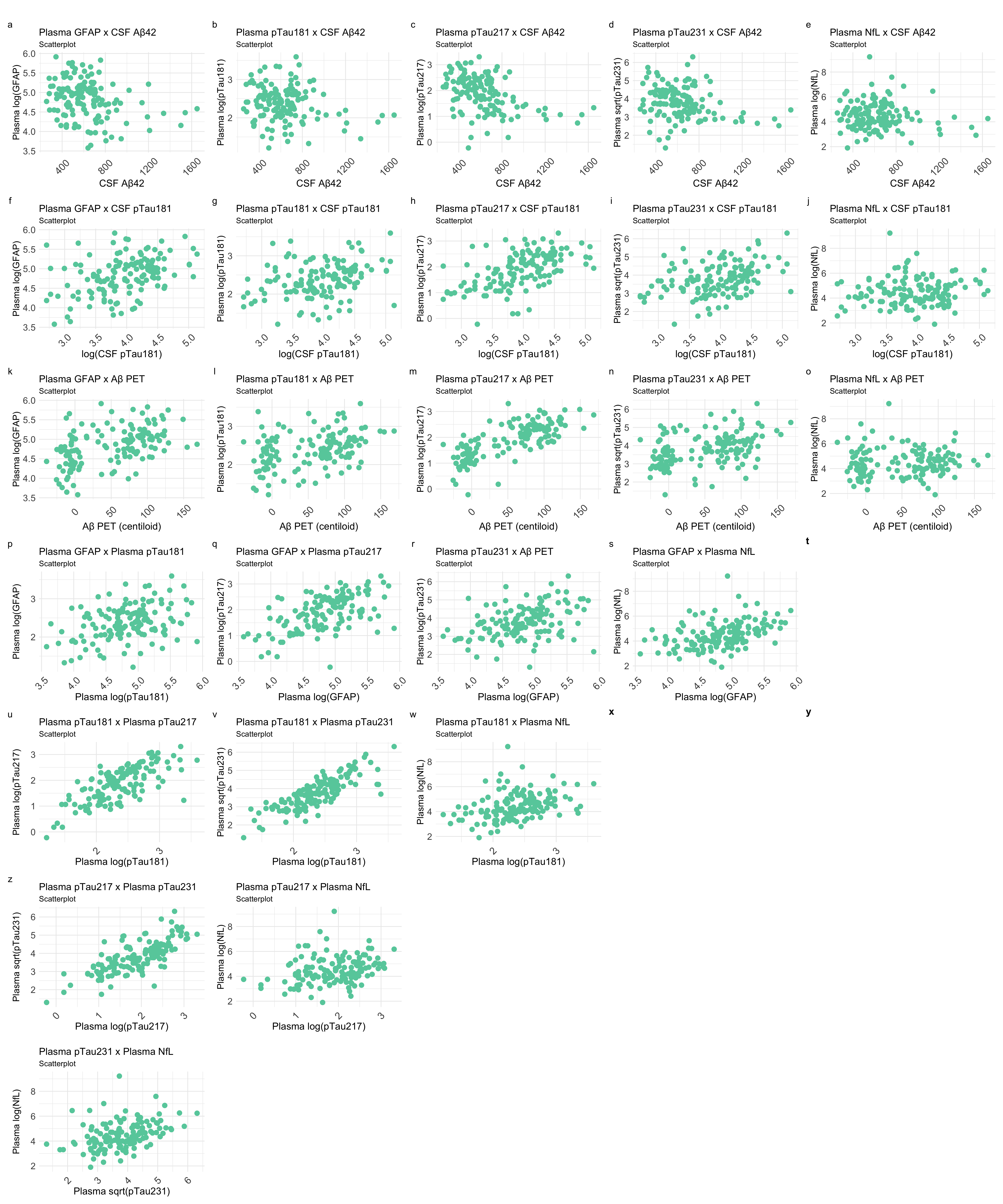
*

Figure S3. Scatterplots of the variables assessed using the Pearson correlation coefficient. Variables that did not meet the assumptions of normal distribution and uniform variance were transformed based on their skewness and tails, either with a logarithmic transformation (plasma GFAP, pTau181, pTau217, NfL, CSF pTau181) or a square root transformation (plasma pTau231).

Table S2: Results of one-way ANOVA

| Plasma biomarker | Predictor | df | F value | Mean Square | *P*-value |
| --- | --- | --- | --- | --- | --- |
| logGFAP | ATN | 5 | 9.60 | 1.70 | < 0.0001 |
| logpTau181 | ATN | 5 | 5.16 | 0.88 | 0.0002 |
| logpTau217 | ATN | 5 | 33.78 | 6.82 | < 0.0001 |
| sqrtpTau231 | ATN | 5 | 6.77 | 4.18 | < 0.0001 |
| logNfL | ATN | 5 | 1.98 | 2.28 | 0.085 |
| logGFAP | Age_group | 3 | 1.04 | 0.25 | 0.38 |
| logpTau181 | Age_group | 3 | 0.84 | 0.17 | 0.47 |
| logpTau217 | Age_group | 3 | 1.52 | 0.69 | 0.21 |
| sqrtpTau231 | Age_group | 3 | 1.72 | 1.27 | 0.17 |
| logNfL | Age_group | 3 | 3.03 | 3.41 | 0.031 |
| logGFAP | Sex | 1 | 7.87 | 1.78 | 0.0058 |
| logpTau181 | Sex | 1 | 0.11 | 0.02 | 0.74 |
| logpTau217 | Sex | 1 | 2.44 | 1.10 | 0.12 |
| sqrtpTau231 | Sex | 1 | 2.27 | 1.69 | 0.13 |
| logNfL | Sex | 1 | 0.19 | 0.22 | 0.66 |
| logGFAP | MMSE_group | 2 | 0.68 | 0.16 | 0.51 |
| logpTau181 | MMSE_group | 2 | 0.31 | 0.06 | 0.74 |
| logpTau217 | MMSE_group | 2 | 0.61 | 0.28 | 0.54 |
| sqrtpTau231 | MMSE_group | 2 | 0.70 | 0.54 | 0.5 |
| logNfL | MMSE_group | 2 | 1.54 | 1.81 | 0.22 |

The MMSE groups were defined as follows: scores of 30-26, 25-20, >19. The age groups were defined as follows: 40-59 years, 60-69 years, 70-79 years, 80-89 years, and 90 years and above. We did not observe significant effects of sex, age, or MMSE group on plasma biomarker levels, except for a small effect of female sex on GFAP and age on NfL. The limited sample size prevented us from including these predictors in the analysis at the sub-ATN level.


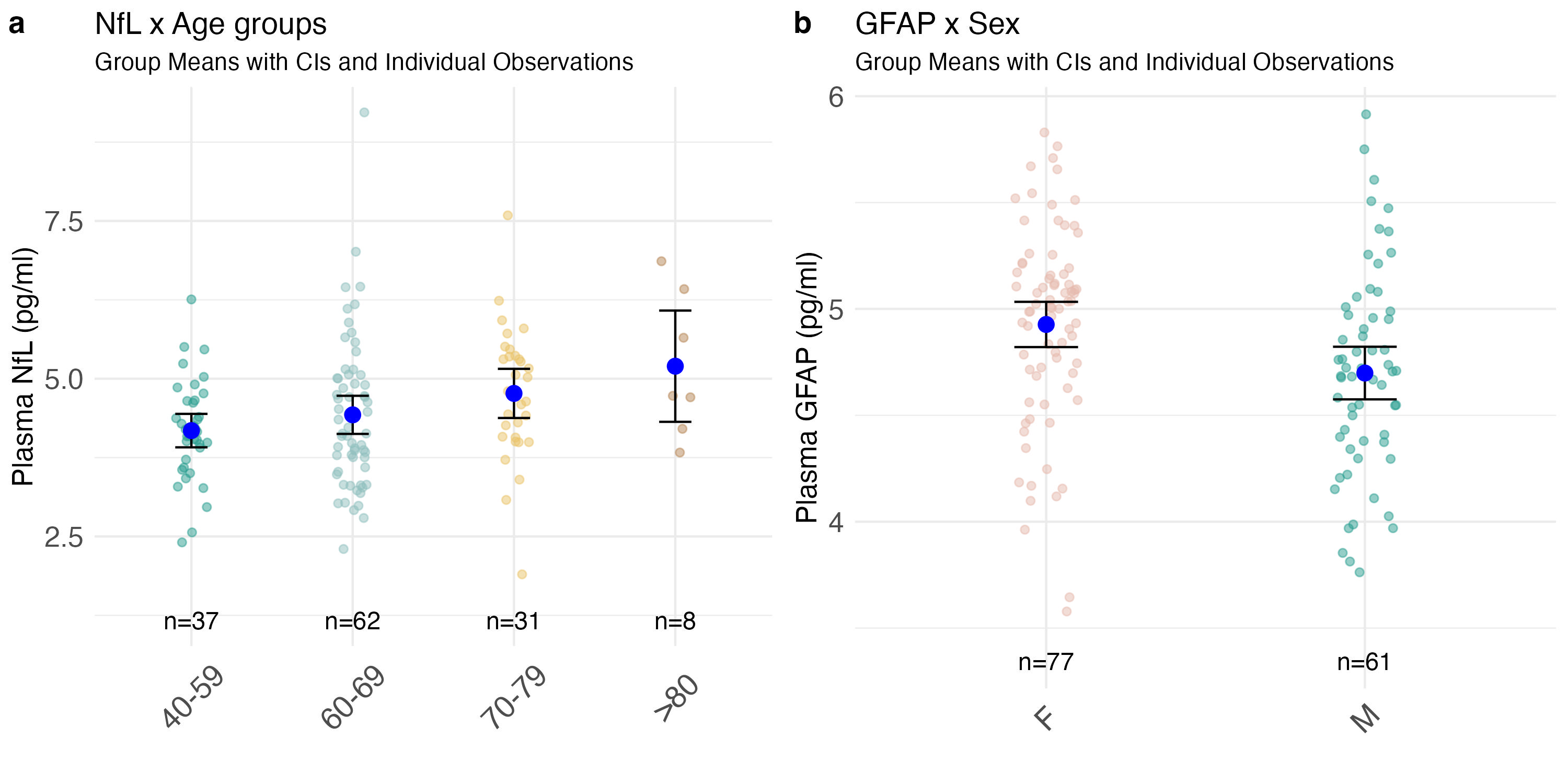


Figure S4. Scatter plots showing mean differences with 95% confidence intervals (CIs) across the groups. **(a)**. The levels of plasma NfL increase with age. **(b)**. The plasma GFAP levels were higher in females.

# Table S3. Partial Correlation Results with confidence intervals

| Variable Pair | Estimate | CI Lower | CI Upper | *P*-value | *P*-value (adjusted) |
| --- | --- | --- | --- | --- | --- |
| logGFAP x logpTau181 | 0.38 | 0.27 | 0.47 | < 0.0001 | < 0.0001 |
| logGFAP x logpTau217 | 0.57 | 0.48 | 0.64 | < 0.0001 | < 0.0001 |
| logGFAP x sqrtpTau231 | 0.38 | 0.28 | 0.48 | < 0.0001 | < 0.0001 |
| logGFAP x logNfL | 0.41 | 0.31 | 0.51 | < 0.0001 | < 0.0001 |
| logGFAP x Aβ PET | 0.49 | 0.40 | 0.58 | < 0.0001 | < 0.0001 |
| logGFAP x CSF_pTau181 | 0.41 | 0.30 | 0.50 | < 0.0001 | < 0.0001 |
| logGFAP x Aβ-42 | -0.28 | -0.39 | -0.17 | < 0.0001 | < 0.0001 |
| logGFAP x Age | 0.07 | -0.04 | 0.19 | 0.21 | 0.23 |
| logpTau181 x logpTau217 | 0.73 | 0.67 | 0.78 | < 0.0001 | < 0.0001 |
| logpTau181 x sqrtpTau231 | 0.82 | 0.78 | 0.85 | < 0.0001 | < 0.0001 |
| logpTau181 x logNfL | 0.36 | 0.25 | 0.46 | < 0.0001 | < 0.0001 |
| logpTau181 x Aβ PET | 0.39 | 0.29 | 0.49 | < 0.0001 | < 0.0001 |
| logpTau181 x CSF_pTau181 | 0.31 | 0.20 | 0.41 | < 0.0001 | < 0.0001 |
| logpTau181 x Aβ-42 | -0.19 | -0.31 | -0.08 | 0.0012 | 0.0016 |
| logpTau181 x Age | 0.07 | -0.05 | 0.18 | 0.27 | 0.28 |
| logpTau217 x sqrtpTau231 | 0.74 | 0.68 | 0.79 | < 0.0001 | < 0.0001 |
| logpTau217 x logNfL | 0.27 | 0.16 | 0.38 | < 0.0001 | < 0.0001 |
| logpTau217 x Aβ PET | 0.76 | 0.70 | 0.80 | < 0.0001 | < 0.0001 |
| logpTau217 x CSF_pTau181 | 0.55 | 0.47 | 0.63 | < 0.0001 | < 0.0001 |
| logpTau217 x Aβ-42 | -0.43 | -0.52 | -0.32 | < 0.0001 | < 0.0001 |
| logpTau217 x Age | -0.11 | -0.22 | 0.01 | 0.071 | 0.091 |
| sqrtpTau231 x logNfL | 0.39 | 0.28 | 0.49 | < 0.0001 | < 0.0001 |
| sqrtpTau231 x Aβ PET | 0.40 | 0.29 | 0.49 | < 0.0001 | < 0.0001 |
| sqrtpTau231 x CSF_pTau181 | 0.37 | 0.26 | 0.47 | < 0.0001 | < 0.0001 |
| sqrtpTau231 x Aβ-42 | -0.24 | -0.35 | -0.13 | < 0.0001 | < 0.0001 |
| sqrtpTau231 x Age | -0.00 | -0.12 | 0.12 | 0.97 | 0.97 |
| logNfL x Aβ PET | 0.03 | -0.08 | 0.15 | 0.56 | 0.58 |
| logNfL x CSF_pTau181 | 0.08 | -0.04 | 0.19 | 0.21 | 0.23 |
| logNfL x Aβ-42 | -0.09 | -0.20 | 0.03 | 0.14 | 0.17 |
| logNfL x Age | 0.29 | 0.18 | 0.40 | < 0.0001 | < 0.0001 |
| Aβ PET x CSF_pTau181 | 0.58 | 0.50 | 0.66 | < 0.0001 | < 0.0001 |
| Aβ PET x Aβ-42 | -0.44 | -0.53 | -0.34 | < 0.0001 | < 0.0001 |
| Aβ PET x Age | -0.08 | -0.20 | 0.04 | 0.18 | 0.21 |
| CSF_pTau181 x Aβ-42 | -0.10 | -0.22 | 0.01 | 0.08 | 0.1 |
| CSF_pTau181 x Age | 0.14 | 0.02 | 0.25 | 0.02 | 0.02 |
| Aβ-42 x Age | 0.23 | 0.12 | 0.34 | 0.0001 | 0.0002 |

Footnote: *P*-values were adjusted using the false discovery rate (FDR) correction method.
